# Readmission After Ischemic Stroke in Ningxia, China, From 2017 to 2021: Retrospective Cohort Study

**DOI:** 10.2196/67522

**Published:** 2025-07-03

**Authors:** Hua Meng, Xingtian Wang, Dongfeng Pan, Xinya Su, Wenwen Lu, Zhuo Liu, Yuhui Geng, Xiaojuan Ma, Ting Pan, Peifeng Liang

**Affiliations:** 1 Hubei Provincial Clinical Research Center for Alzheimer's Disease, Tianyou Hospital, School of Medicine, Wuhan University of Science and Technology, Wuhan, China; 2 Brain Science and Advanced Technology Institute, Wuhan University of Science and Technology, Wuhan, China; 3 Medical Record Statistics Department, General hospital of Ningxia Medical University, Yinchuan, China; 4 Department of Emergency Medicine, People's Hospital of Ningxia Hui Autonomous Region, Ningxia Medical University, Yinchuan, China; 5 School of Public Health, Ningxia Medical University, Yinchuan, China; 6 Ningxia Key Laboratory of Environmental Factors and Chronic Disease Control, Yinchuan, China; 7 Futian Center for Chronic Disease Control, Shenzhen, China; 8 Department of Medical Affair, People's Hospital of Ningxia Hui Autonomous Region, Ningxia Medical University, 301 Zhengyuan North Street, Yinchhuan, 750002, China, 86 13895085519

**Keywords:** ischemic stroke, rehospitalization, Least Absolute Shrinkage and Selection Operator, stabilized inverse probability of treatment weighting, cox proportional hazards regression model

## Abstract

**Background:**

Stroke remains a major cause of death and disability worldwide. Ischemic stroke is the most common type of stroke. Readmissions after hospitalization increase the patient burden and waste health resources.

**Objective:**

This study aimed to calculate rehospitalization rates and explore risk factors associated with rehospitalization in ischemic stroke.

**Methods:**

In this retrospective cohort study, we identified 12,782 patients admitted for ischemic stroke at People’s Hospital of Ningxia Hui Autonomous Region between January 2017 and December 2021. Groups were determined based on the ID number. The most important factors were selected using the Least Absolute Shrinkage and Selection Operator regression model. Stabilized inverse probability of treatment weighting (SIPTW) was used to correct baseline imbalances between groups. The adjusted hazard ratios and Kaplan-Meier survival curves of significant factors after SIPTW were calculated using stepwise backward Cox regression.

**Results:**

A total of 10,727 patients were included in the study. Among them, 12.7% and 7.2% were readmitted within 5 years and 1 year, respectively. Stepwise backward Cox analysis of SIPTW showed that diabetes was the influencing factor for rehospitalization within 5 years (1.15, 1.02‐1.30) and 1 year (1.21, 1.03‐1.43). Additionally, the female gender was identified as a protective factor against readmission within 5 years (0.83, 0.74‐0.93).

**Conclusions:**

Although the rate of rehospitalization varied among patients with ischemic stroke at different time points, the significant factors remained consistent. Therefore, early prevention and treatment methods may be consistent.

## Introduction

Because of its high mortality and disability rate, cerebrovascular disease (CVD) seriously threatens human health and brings great pressure to the medical care system, especially in limited-income countries [1,2]. CVD is the primary cause of death and disability in adults in China [3,4]. It is estimated that about 330 million patients experience difficulties from CVD in China [5]. The Annual Report on Cardiovascular Health and Diseases in China (2021) shows that, in 2019, the total number of discharged patients with CVD was 26.8441 million. The total hospitalization expenses of CVD were RMB 136.028 billion. After adjusting for price factors, the average annual growth rate of total hospitalization expenses for ischemic stroke and hemorrhagic stroke has been 18.82% and 13.51%, respectively, since 2004 [6]. Readmissions are common: up to 22% of individuals experience 30-day readmissions after neurologic hospitalization [7].

High readmission rates may indicate unresolved problems at initial discharge [8], the quality of immediate post-hospital care, a more chronically ill population, or combinations of these factors. High readmission rates are also associated with a substantial economic burden on the health care system and may represent opportunities to reduce avoidable costs [9]. Reduction of readmission rates has become the goal of national health care reform, health insurance, and Medicaid service centers.

To reduce rehospitalization rates in patients with ischemic stroke, it is essential to fully understand preventable and unpreventable predictors that may influence rehospitalization. Previous studies have reported that infection [10], advanced age [11], and diabetes [12] are the most common causes of rehospitalization in patients with stroke. Furthermore, because most readmissions were measured within 30 days of the event, it is unknown whether the reasons for long-term readmissions differ [13].

This study focused on 3 main areas to better understand the factors contributing to rehospitalization in patients with ischemic stroke: the rates of rehospitalization at various intervals (1 year and 5 years), the differences in patient characteristics between those who were rehospitalized and those who were not, and the factors influencing rehospitalization in patients.

## Methods

### Ethical Considerations

This study followed the principles of the Declaration of Helsinki and was approved by People's Hospital of Ningxia Hui Autonomous Region (2020-KY-053), and the informed consent was waived off by the review boards due to the nature of this research. All data in the manuscript and supplementary materials were anonymized in accordance with ethical standards, ensuring no personally identifiable information could be discerned.

### Data Resources and Patients

This is a retrospective cohort study. In 2022, we examined electronic health record data from all patients who were discharged from People’s Hospital of Ningxia Hui Autonomous Region between January 2017 and December 2021. The electronic health record data were anonymized and accessed in a secure environment. The data have a hierarchical structure, including medical record number, demographic characteristics, primary and secondary diagnoses, procedures, method of payment, and a total of 642 variables. The hospital enforces rigorous follow-up and intervention protocols for patients with stroke. Qualified specialists in brain and heart health provide guidance on exercise and rehabilitation after discharge via the WeChat app. Public health physicians conduct telephone follow-ups at 3 and 6 months post-discharge, while a professional doctor performs an in-person visit at 12 months. This visit primarily includes blood sample testing, carotid ultrasound examinations, and adjustments to the patient’s medication regimen.

The criteria for data included in this study were as follows: (1) patients aged 18 years or older with a principal diagnosis of ischemic stroke (International Classification of Diseases, Tenth Edition [ICD]-10: I63); (2) patients readmitted to any hospital after discharge due to an ischemic stroke-related condition. The first onset of ischemic stroke was considered the index event, and readmission was the endpoint event. Patients discharged from the hospital who were deceased, under 18 years of age, or had incorrect ID numbers and encoding formats were excluded. If the same patient had multiple rehospitalization records, only the first 2 hospitalization records were retained.

### Outcome Measures

Rehospitalization for ischemic stroke refers to the same individual being readmitted to the hospital with ischemic stroke as the primary diagnosis. The rehospitalization rate was calculated by dividing the number of patients readmitted after hospitalization by the total number of patients discharged alive during the same period [14].

Rehospitalizations were identified using ID numbers and the main diagnostic code for the disease from the electronic health data of The People’s Hospital of Ningxia Hui Autonomous Region from 2017 to 2021. The primary outcome was a 5-year rehospitalization rate, and the secondary outcomes were 1-year rehospitalization rates.

### Study Variables

Published relevant literature was reviewed to summarize and integrate the factors involved [7,9,11,12,15-20]. Covariates considered for confounding adjustment included sex, age (<60, 60‐69, 70‐79, ≥80), admission route, length of stay (LOS), anemia (D50-64), thyroid disease (E00-07), diabetes (E10-14), dementia (F00-03), parkinsonism (G20), transient ischemic attack and related syndrome (G45), hypertension (I10-15), coronary heart disease (CHD) (I20-25), paroxysmal tachycardia (I47), atrial fibrillation and flutter (I48), heart failure (I50), arteriosclerosis (I70), embolism and thrombosis (I74), acute upper respiratory tract infection (J00-06), pneumonia (J12-18), renal failure (N17-N19), and urinary tract infection (N39.000), treatment (anticoagulants, thrombectomy, thrombolysis, thrombectomy and thrombolysis), and NIH Stroke Scale (NIHSS) score.

### Statistical Analysis

The variables of admission route and NIHSS score had missing values; the number of missing values was 5 (0.04%) and 10346 (96.4%), respectively. The missing mechanism was determined to be missing completely at random according to the correlation coefficient matrix between the missing values and other variables (Table S1 in Multimedia Appendix 1) [21]. Therefore, the incomplete data for admission route and NIHSS score were imputed simultaneously using multiple imputations (n=25) with the R package MICE (TNO and University of Twente) [22,23]. Based on the Akaike information criterion (AIC) value, one of the imputed datasets was selected for analysis.

Some previous studies have demonstrated that the Least Absolute Shrinkage and Selection Operator (LASSO) method was superior to traditional methods [24-26]. LASSO regression was used to avoid overfitting and collinearity [26]. Therefore, LASSO analysis was used to select variables to be included in the Cox proportional hazards regression model (Cox model). The “glmnet” package was used to analyze the LASSO regression model [27].

Stabilized inverse probability of treatment weighting (SIPTW) was used to achieve a balanced comparison between the readmission and non-readmission groups. This method helps maintain the original data’s sample size and ensures an appropriate class I error rate [28]. Probability was estimated through a logistic regression model with rehospitalization as the dependent variable, considering variables such as age, anemia, hypertension, CHD, length of hospital stay, treatment, urinary tract infection, and NIHSS [29]. The balance of potential confounders at baseline was evaluated using the absolute standardized difference (SMD), where an SMD greater than 0.1 indicated a significant difference in potential confounders between cases and controls [30].

The Cox model was constructed by backward Cox regression using the AIC selection criteria, and the best model was chosen based on the least AIC [31-33]. The “survival” package was used for Cox analysis, and the “MASS” package was used to perform stepwise backward analysis. The log-rank test conducted stepwise backward Cox analysis of significant factors for rehospitalization rates for SIPTW. The Kaplan-Meier curve was obtained using the “survminer” package.

### Model Evaluation

Calibration curves were plotted using the “rms” package, which compares the agreement between the model’s predicted and observed probabilities [34].

Decision curve analysis was conducted using the ‘ggDCA’ package to assess the utility of a model in supporting clinical decisions [35].

Clinical impact curves were drawn using the ‘rmda’ package to evaluate the model’s recognition value in rehospitalized patients [36].

A 2-sided *P* value of less than .05 indicated statistical significance. Data screening and extraction were performed using Excel version 2016, and other analyses were carried out using R (version 4.2.2).

## Results

**Figure 1.**
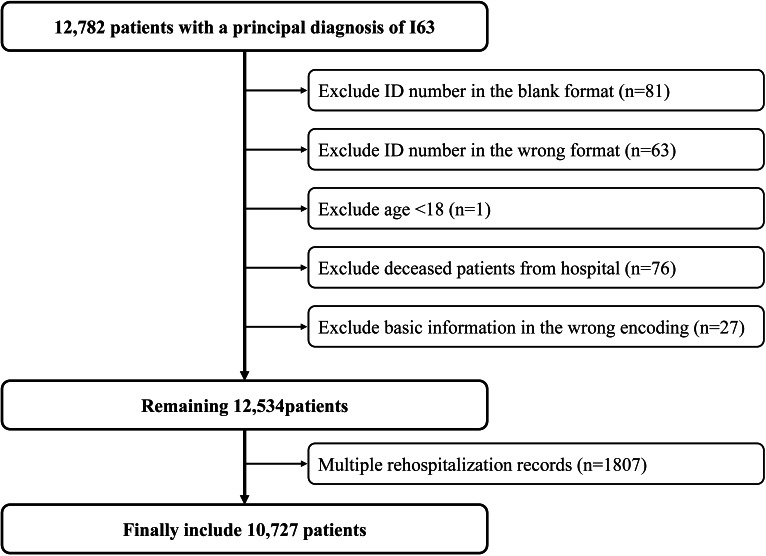
Flowchart of patient selection for this study.

**Figure 2.**
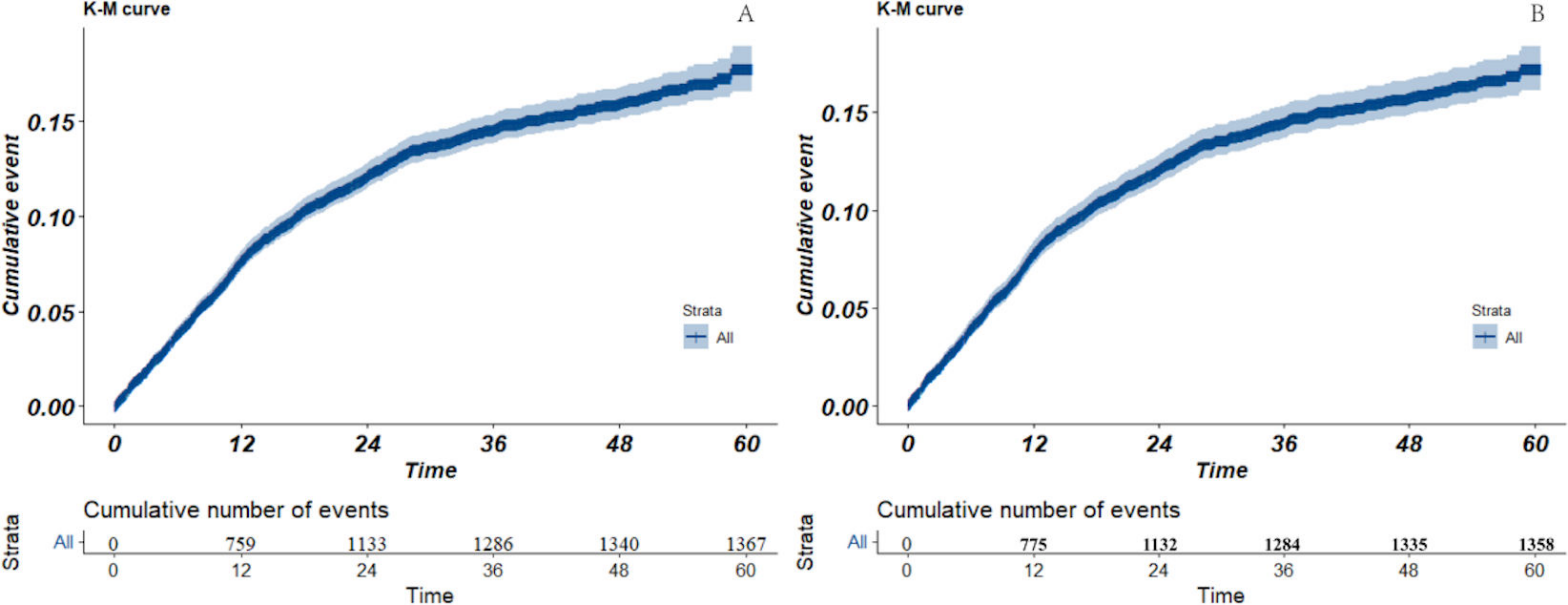
Kaplan-Meier curve of rehospitalization patients. A: cohort data; B: stabilized inverse probability of treatment weighting data. K-M curve: Kaplan-Meier curve.

The results of the data inclusion procedure are shown in Figure 1. A total of 10,727 eligible patients with ischemic stroke were included in the study. Among all patients, 12.7% (1367) were readmitted within 5 years after discharge, and 7.2% (769) were rehospitalized within 1 year. The Kaplan-Meier curves are shown in Figure 2.

There was a significant imbalance in age, treatment, anemia, hypertension, CHD, and LOS between the 2 groups with or without rehospitalization within 5 years. After using the SIPTW, the SMD did not exceed 0.1. Baseline characteristics are shown in Table 1. Significant differences in age, anemia, hypertension, CHD, diabetes, LOS, and NIHSS were observed between the 2 groups with or without rehospitalization within 1 year. Following SIPTW adjustment, balance was achieved between the 2 groups (Table S2 in Multimedia Appendix 1).

**Figure 3.**
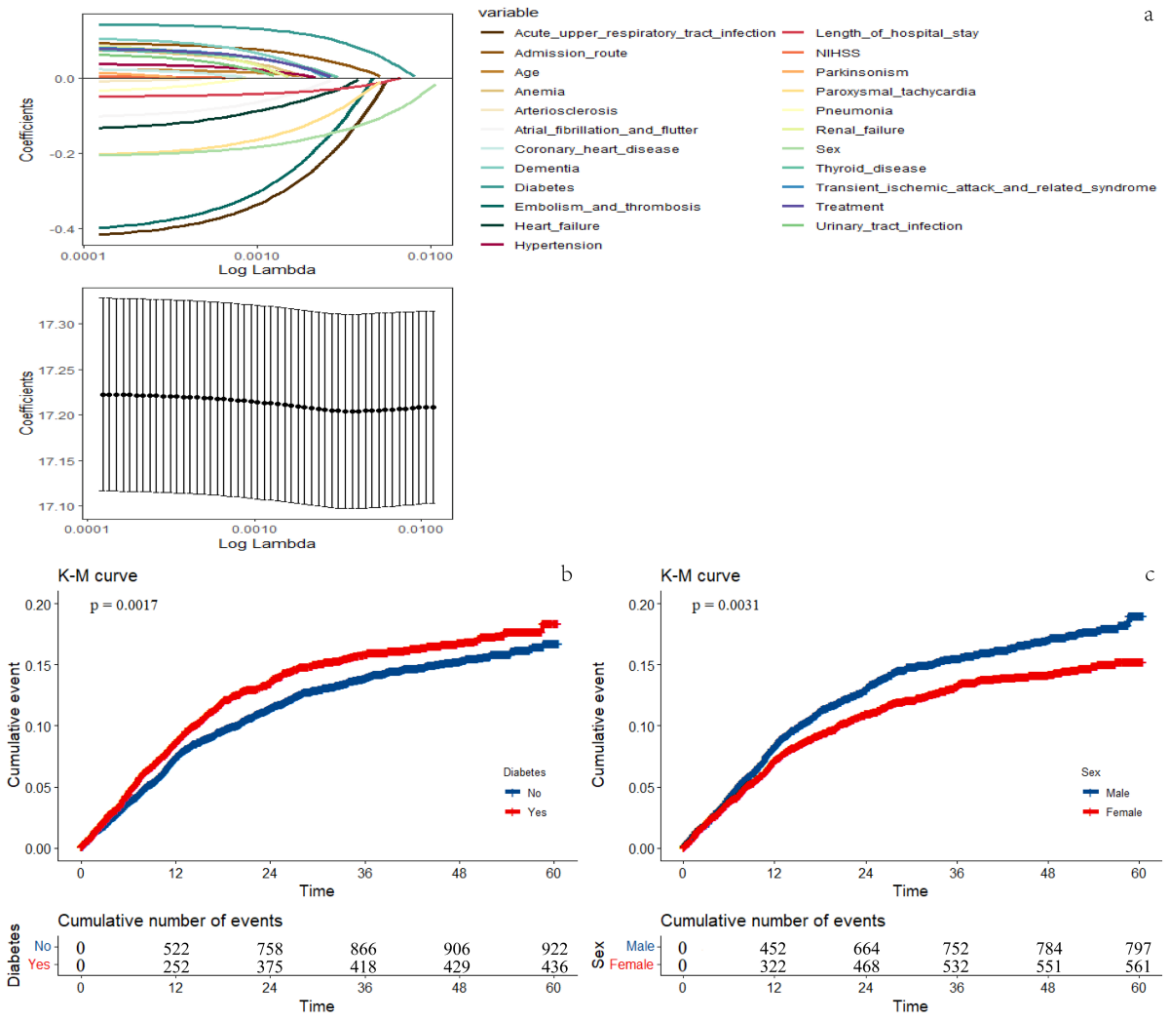
Stabilized inverse probability of treatment weighting and Least Absolute Shrinkage and Selection Operator (LASSO) regression, Kaplan-Meier (K-M) curves of rehospitalization within 5 years in patients with ischemic stroke (a): LASSO regression; (b) within 5 years in patients with ischemic stroke without or with diabetes; and (c) within 5 years in different sex patients with ischemic stroke. NIHSS: NIH Stroke Scale.

On readmission within 5 years, sex, admission route, diabetes, heart failure, acute upper respiratory tract infection, paroxysmal tachycardia, LOS, embolism, and thrombosis were indicated for inclusion from a 5-year SIPTW LASSO regression. Analyzing the above variables mentioned above, stepwise backward Cox regression after SIPTW showed that the significant variable was diabetes, with a hazard ratio (HR) of 1.15 and a 95% confidence interval (95% CI) of 1.02 to 1.30. As shown in Figure 3, among 3175 patients with ischemic stroke with diabetes, 436 (13.7%) patients with ischemic stroke and diabetes were rehospitalized for treatment within five years. Sex (HR 0.83; 95% CI 0.74‐0.93) was identified as a protective factor for rehospitalization in patients with ischemic stroke (Table 2). The Kaplan-Meier curves are displayed in Figure 3(B and C).

Sex, diabetes, paroxysmal tachycardia, and urinary tract infection were indicated for inclusion from a 1-year SIPTW LASSO regression (Figure S1 in Multimedia Appendix 1). In the stepwise backward Cox analysis of SIPTW, the significant factor was diabetes (HR 1.21; 95% CI 1.03‐1.43).

Unweighted stepwise backward Cox analyses are listed in Table S3 in Multimedia Appendix 1.

**Table 1.** Characteristics between readmission and no readmission groups within 5 years.

	Cohort data				SIPTW^a^ data			
	No readmission	Readmission	*P* value	SMD	No readmission	Readmission	*P* value	SMD
	N=9360	N=1367			N=9360.8	N=1358.1		
Male (n, %)	5159 (55.1)	794 (58.1)	.039	0.060	5148.3 (55.0)	797.0 (58.7)	.020	0.075
Age, years (n, %)								
<60	2267 (24.2)	227 (16.6)	<.001	0.211	2175.2 (23.2)	305.5 (22.5)	.863	0.021
60‐69	2650 (28.3)	375 (27.4)			2639.6 (28.2)	388.8 (28.6)		
70‐79	2911 (31.1)	481 (35.2)			2960.1 (31.6)	427.1 (31.5)		
≥80	1532 (16.4)	284 (20.8)			1585.8 (16.9)	236.6 (17.4)		
Admission route (n, %)								
Emergency	4579 (48.9)	629 (46.0)	.187	0.060	4567.3 (48.8)	625.7 (46.1)	.259	0.065
Others	1 (0.0)	0 (0.0)			1.0 (0.0)	0.0 (0.0)		
Outpatient	4768 (51.0)	736 (53.8)			4780.2 (51.1)	728.4 (53.6)		
Transferred	12 (0.1)	2 (0.1)			12.2 (0.1)	3.9 (0.3)		
Treatment (n, %)								
Anticoagulants	8847 (94.5)	1328 (97.1)	<.001	0.133	8879.2 (94.9)	1295.3 (95.4)	.931	0.028
Thrombectomy	19 (0.2)	1 (0.1)			17.5 (0.2)	2.7 (0.2)		
Thrombectomy and thrombolysis	38 (0.4)	2 (0.1)			34.9 (0.4)	3.7 (0.3)		
Thrombolysis	456 (4.9)	36 (2.6)			429.2 (4.6)	56.4 (4.2)		
Disease (n, %)
Parkinsonism (n, %)	111 (1.2)	25 (1.8)	.047	0.053	112.8 (1.2)	18.5 (1.4)	.586	0.014
Anemia (n, %)	367 (3.9)	28 (2.0)	.001	0.110	344.9 (3.7)	52.9 (3.9)	.786	0.011
Thyroid disease (n, %)	1249 (13.3)	190 (13.9)	.574	0.016	1253.0 (13.4)	190.6 (14.0)	.550	0.019
Dementia (n, %)	184 (2.0)	33 (2.4)	.272	0.031	186.5(2.0)	33.5 (2.5)	.300	0.032
Transient ischemic attack and related syndrome (n, %)	643 (6.9)	108 (7.9)	.163	0.039	647.1 (6.9)	111.2 (8.2)	.129	0.048
Hypertension (n, %)	6606 (70.6)	1045 (76.4)	<.001	0.133	6677.2 (71.3)	973.1 (71.7)	.835	0.007
Coronary heart disease (n, %)	2132 (22.8)	383 (28.0)	<.001	0.121	2196.0 (23.5)	327.4 (24.1)	.616	0.015
Paroxysmal tachycardia (n, %)	660 (7.1)	68 (5.0)	.004	0.087	668.3 (7.1)	65.3 (4.8)	.003	0.099
Diabetes (n, %)	2706 (28.9)	446 (32.6)	.005	0.081	2738.5 (29.3)	435.6 (32.1)	.052	0.061
Atrial fibrillation and flutter (n, %)	564 (6.0)	72 (5.3)	.267	0.033	570.5 (6.1)	67.4 (5.0)	.124	0.050
Heart failure (n, %)	735 (7.9)	122 (8.9)	.172	0.039	758.5 (8.1)	102.2 (7.5)	.476	0.022
Arteriosclerosis (n, %)	5190 (55.4)	812 (59.4)	.006	0.080	5221.6 (55.8)	792.1 (58.3)	.113	0.051
Embolism and thrombosis (n, %)	165 (1.8)	18 (1.3)	.234	0.036	167.3 (1.8)	14.8 (1.1)	.074	0.059
Acute upper respiratory tract infection (n, %)	127 (1.4)	11 (0.8)	.091	0.053	128.5 (1.4)	11.9 (0.9)	.192	0.047
Pneumonia (n, %)	309 (3.3)	43 (3.1)	.763	0.009	315.1 (3.4)	47.2 (3.5)	.869	0.006
Renal failure (n, %)	148 (1.6)	20 (1.5)	.742	0.010	148.7 (1.6)	21.0 (1.5)	.915	0.004
Urinary tract infection (n, %)	324 (3.5)	32 (2.3)	.031	0.067	310.4 (3.3)	42.8 (3.2)	.815	0.009
Length of hospital stay (n, %)								
<Q1 (<8)	2394 (25.6)	162 (11.9)	<.001	0.414	2229.9 (23.8)	311.2 (22.9)	.545	0.023
Q1-Q2 (8‐10)	2029 (21.7)	262 (19.2)			1999.3 (21.4)	291.2 (21.4)		
Q2-Q3 (10‐13)	2728 (29.1)	440 (32.2)			2764.9 (29.5)	410.7 (30.2)		
≥Q3 (≥13)	2209 (23.6)	503 (36.8)			2366.7 (25.3)	344.9 (25.4)		
NIHSS (n, %)								
Q1 (≤1)	2459 (26.3)	389 (28.5)	.008	0.100	2483.9 (26.5)	351.6 (25.9)	.476	0.023
Q1-Q2 (1‐2)	2766 (29.6)	361 (26.4)			2728.6 (29.1)	396.8 (29.2)		
Q2-Q3 (2‐4)	2125 (22.7)	348 (25.5)			2159.7 (23.1)	325.1 (23.9)		
>Q3 (>4)	2010 (21.5)	269 (19.7)			1988.5 (21.2)	284.6 (21.0)		

a SIPTW: stabilized inverse probability of treatment weighting.

**Table 2.** Stepwise backward COX regression after SIPTW^a^.

	B	SE	*P* Value	HR^b^	Lower limit	Upper limit
5 years						
Sex (female)	−0.188	0.055	.002	0.83	0.74	0.93
Diabetes (yes)	0.140	0.058	.027	1.15	1.02	1.30
Embolism and thrombosis (yes)	−0.388	0.262	.155	0.68	0.40	1.16
Paroxysmal tachycardia (yes)	−0.209	0.127	.130	0.81	0.62	1.06
Acute upper respiratory tract infection (yes)	−0.445	0.291	.185	0.64	0.33	1.24
1 year						
Sex (female)	−0.135	0.073	.096	0.87	0.74	1.02
Diabetes (yes)	0.192	0.077	.023	1.21	1.03	1.43
Paroxysmal tachycardia (yes)	−0.319	0.168	.107	0.73	0.49	1.07

a SIPTW: stabilized inverse probability of treatment weighting.

b HR: hazard ratio.

### Model Validation

**Figure 4.**
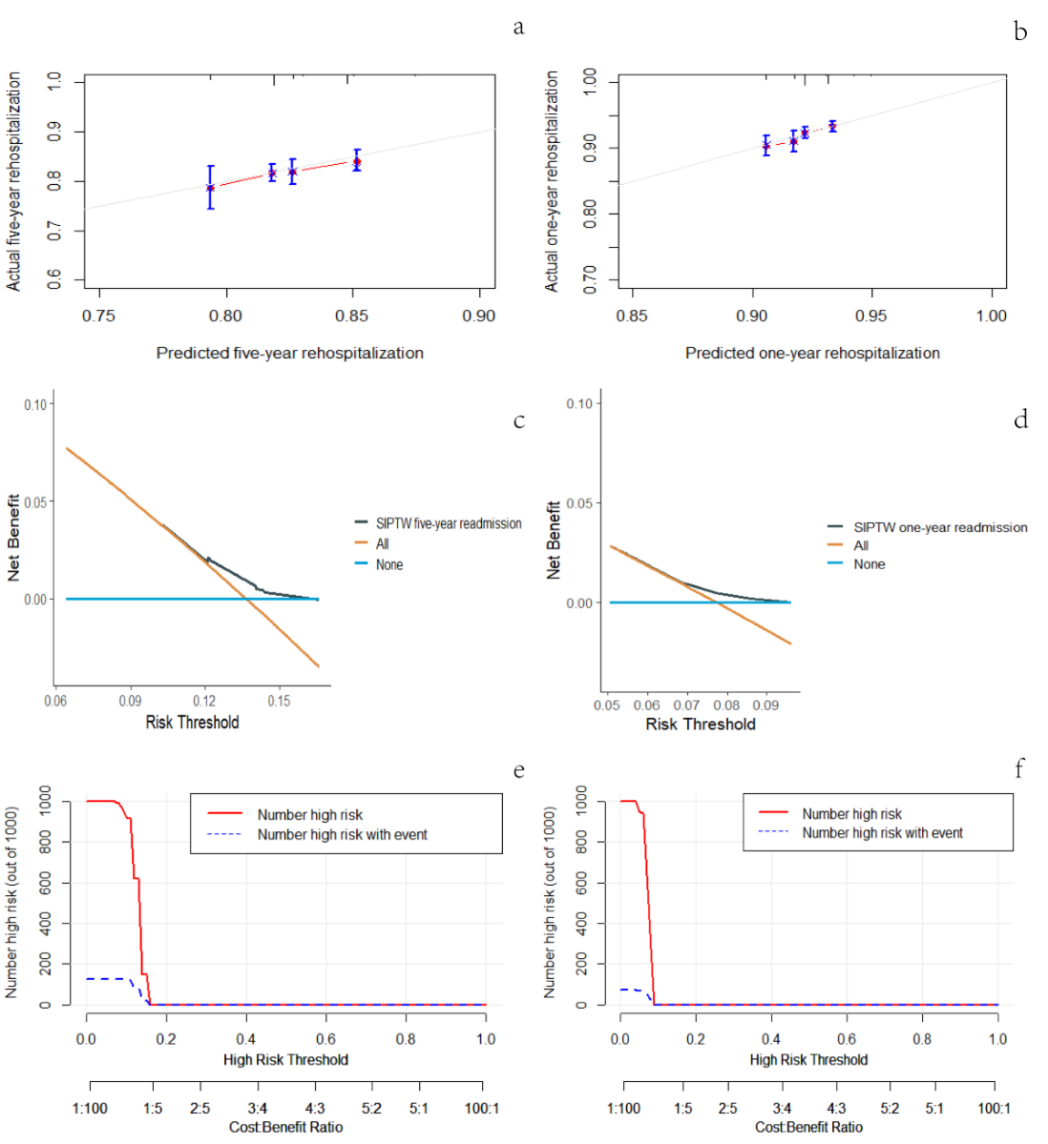
Model evaluation of results from 5-year and 1-year rehospitalization analysis after SIPTW (a, b: calibration curve; c, d: decision curve analysis; e, f: clinical impact curve. SIPTW: stabilized inverse probability of treatment weighting.

After performing SIPTW and stepwise backward Cox analysis, it can be observed from Figure 4(A and B) that the calibration curve of the model closely aligns with the diagonal line, suggesting that the model has good predictive power.

The decision curve analysis of the model indicated that if the patient’s risk threshold probability for rehospitalization within 5 years was between 0.074 and 0.165 (Figure 4C), and within 1 year was between 0.057 and 0.096 (Figure 4D), then using the model to determine the need for rehospitalization offers added advantages over the options of full rehospitalization or no rehospitalization.

The clinical impact curve of the model showed that when the risk threshold was greater than 0.13, the high-risk rehospitalization cases of ischemic stroke predicted by the model within 5 years closely matched the true ischemic stroke rehospitalized cases (Figure 4E). When the threshold was above 0.07, the high-risk rehospitalization cases of ischemic stroke predicted by the model within 1 year closely resembled the true ischemic stroke rehospitalized cases (Figure 4F).

## Discussion

### Principal Findings and Comparison With Previous Works

In this study, there were similarities and differences in the influencing factors of rehospitalization at different times. The SIPTW and unweighted stepwise Cox analyses found gender to be significant among the LASSO regression factors for rehospitalization within 5 years.

A study in Sichuan Province included 1,066,752 patients with stroke with an average follow-up of 1.15 years, with a rehospitalization rate of 23% [37]. Between 1986 and 2001, there were 128,511 stroke hospitalizations in Scotland, and approximately 10.8% of patients were rehospitalized within 5 years due to stroke [38]. A study in Singapore included 12,559 patients with stroke, and the rehospitalization rate for recurrent stroke within 5 years was approximately 18.4% [39]. In our study, the low rate of rehospitalization may be attributed to the effective communication between medical staff and patients and their families upon discharge [40], which improved patient compliance with treatment and the professionalism of the accompanying staff. On the other hand, participants were from a single hospital, and in cases of acute illness, they may opt for medical care from nearby facilities like community general practitioners or other urban areas in Ningxia or other provinces [41]. Additionally, these patients are typically managed by specialized palliative care teams and may choose not to be readmitted in the event of complications, opting instead for care at the palliative care facility. The COVID-19 pandemic also had an indirect impact on readmission rates, which is primarily reflected in the reduced accessibility of medical resources and changes in patient health care-seeking behavior. Our study did not systematically collect data on telemedicine usage during the pandemic, which may result in a potential underestimation of readmission rates. Future research should incorporate indicators of health care resource utilization to more comprehensively evaluate the long-term effects of the pandemic on the management of ischemic stroke.

Relevant meta-analyses have shown that common patient-related risk factors associated with increased readmission rates include age, heart failure, nephropathy, respiratory disease, peripheral arterial disease, and diabetes [42]. This aligns with the variables initially screened by LASSO regression in this study. In this study, the results of weighted stepwise Cox regression analysis and unweighted stepwise Cox regression analysis are inconsistent due to variations in the distribution of these variables between the non-rehospitalization group and the rehospitalization group in the original cohort data. Through the SIPTW method used in this study, the original dataset is preserved while adjusting for individual differences between groups, leading to a more balanced data distribution and more reliable results [43].

After adjusting for confounding variables, the study found that women patients had a lower risk of re-hospitalization within 5 years. In the analysis of both 5-year rehospitalization and 1-year rehospitalization, diabetes was considered a risk factor, which aligns with findings from previous studies [19,39,44]. This indicates that readmissions are more prevalent among men and individuals with diabetes, leading to higher health care costs [45]. Patients with diabetes exhibit a more pronounced procoagulant state [46] and experience delayed reperfusion of the ischemic penumbra [47], resulting in poorer recovery for patients with stroke. Experimental evidence suggests that hyperglycemia reduces the number of protective non-inflammatory macrophages, thereby increasing mediators of ischemic brain injury [48] and disrupting the blood-brain barrier [49], impacting the prognosis of patients with ischemic stroke.

### Strengths

The main strength of this study is that we investigated patients with ischemic stroke at the People’s Hospital of Ningxia Hui Autonomous Region over a 5-year period. We conducted follow-ups using electronic medical records from multiple hospitals. We included comprehensive details from the initial page of each patient’s medical record for a comparative summary. Additionally, we used LASSO regression to screen factors, used SIPTW to adjust for confounding variables between groups, and conducted backward stepwise Cox analysis to refine the model.

### Limitations

This study also has some potential limitations. First, we identified study subjects using ICD-10 diagnosis codes in the database. However, the coding accuracy may vary depending on the complexity of the disease or institution [50]. Second, risk adjustment may be inadequate due to limited information on the case’s front page and a lack of clinical details, such as the disease’s severity and death information. Laboratory tests for atmospheric environmental indicators are missing. Recent studies have suggested that neutrophil percentage, red blood cell distribution width, alkaline phosphatase [51], and ambient particulate matter pollution of different sizes (PM_1_, PM_2.5_, and PM_10_) [37] influence the readmission of patients with stroke prognosis after discharge. Third, the data came from only 1 hospital, and the findings may not represent other geographical areas or medical institutions, limiting the universality of the results. However, the results of the preliminary screening of factors in this study align with previous research results in different regions. Finally, our study primarily focuses on outcomes during hospitalization and mid- to long-term outcomes. However, it does not include long-term follow-up data after discharge, which limits a comprehensive assessment of patients’ long-term functional recovery, risk of recurrence, and quality of life.

### Conclusions

In conclusion, our findings suggest inconsistent rehospitalization rates for ischemic stroke disease at different times and that the same factors influence rehospitalization rates. Early prevention and treatment of influencing factors are required for the prognosis of ischemic stroke. Future studies should evaluate whether targeted interventions in populations with high-risk factors reduce rehospitalization rates.

## Supplementary material

10.2196/67522 Multimedia Appendix 1 Supplementary tables and figure.

## Abbreviations

AIC: Akaike information criterion
CHD: coronary heart disease
CVD: cerebrovascular disease
ICD: International Classification of Diseases
LASSO: Least Absolute Shrinkage and Selection Operator
LOS: length of stay
NIHSS: NIH Stroke Scale
SIPTW: stabilized inverse probability of treatment weighting
SMD: standardized difference

## References

[1] Roth GA, Abate D, Abate KH (2018). Global, regional, and national age-sex-specific mortality for 282 causes of death in 195 countries and territories, 1980-2017: a systematic analysis for the Global Burden of Disease Study 2017. Lancet.

[2] Katan M, Luft A (2018). Global burden of stroke. Semin Neurol.

[3] Ma LY, Chen WW, Gao RL (2020). China cardiovascular diseases report 2018: an updated summary. J Geriatr Cardiol.

[4] Wang YJ, Li ZX, Gu HQ (2022). China Stroke Statistics: an update on the 2019 report from the National Center for Healthcare Quality Management in Neurological Diseases, China National Clinical Research Center for Neurological Diseases, the Chinese Stroke Association, National Center for Chronic and Non-communicable Disease Control and Prevention, Chinese Center for Disease Control and Prevention and Institute for Global Neuroscience and Stroke Collaborations. Stroke Vasc Neurol.

[5] (2021). Interpretation of Annual Report on Cardiovascular Health and Diseases in China 2019. Cardiology Discovery.

[6] Writing committee of the report on cardiovascular health and diseases in China (2022). Report on cardiovascular health and diseases in China 2021: an updated summary. Biomed Environ Sci.

[7] Guterman EL, Douglas VC, Shah MP, Parsons T, Barba J, Josephson SA (2016). National characteristics and predictors of neurologic 30-day readmissions. Neurology (ECronicon).

[8] Kilkenny MF, Longworth M, Pollack M (2013). Factors associated with 28-day hospital readmission after stroke in Australia. Stroke.

[9] Lichtman JH, Leifheit-Limson EC, Jones SB (2010). Predictors of hospital readmission after stroke: a systematic review. Stroke.

[10] Bjerkreim AT, Thomassen L, Waje-Andreassen U, Selvik HA, Næss H (2016). Hospital readmission after intracerebral hemorrhage. J Stroke Cerebrovasc Dis.

[11] Bjerkreim AT, Thomassen L, Brøgger J, Waje-Andreassen U, Næss H (2015). Causes and predictors for hospital readmission after ischemic stroke. J Stroke Cerebrovasc Dis.

[12] Rumalla K, Smith KA, Arnold PM, Mittal MK (2018). Subarachnoid hemorrhage and readmissions: national rates, causes, risk factors, and outcomes in 16,001 hospitalized patients. World Neurosurg.

[13] Smith MA, Liou JI, Frytak JR, Finch MD (2006). 30-Day survival and rehospitalization for stroke patients according to physician specialty. Cerebrovasc Dis.

[14] Jencks SF, Williams MV, Coleman EA (2009). Rehospitalizations among patients in the Medicare fee-for-service program. N Engl J Med.

[15] Ottenbacher KJ, Graham JE, Ottenbacher AJ (2012). Hospital readmission in persons with stroke following postacute inpatient rehabilitation. J Gerontol A Biol Sci Med Sci.

[16] Burke JF, Skolarus LE, Adelman EE, Reeves MJ, Brown DL (2014). Influence of hospital-level practices on readmission after ischemic stroke. Neurology (ECronicon).

[17] Nouh AM, McCormick L, Modak J, Fortunato G, Staff I (2017). High mortality among 30-day readmission after stroke: predictors and etiologies of readmission. Front Neurol.

[18] Bjerkreim AT, Naess H, Khanevski AN, Thomassen L, Waje-Andreassen U, Logallo N (2019). One-year versus five-year hospital readmission after ischemic stroke and TIA. BMC Neurol.

[19] Qiu X, Xue X, Xu R (2021). Predictors, causes and outcome of 30-day readmission among acute ischemic stroke. Neurol Res.

[20] Loebel EM, Rojas M, Wheelwright D, Mensching C, Stein LK (2022). High risk features contributing to 30-day readmission after acute ischemic stroke: a single center retrospective case-control study. Neurohospitalist.

[21] Sterne JAC, White IR, Carlin JB (2009). Multiple imputation for missing data in epidemiological and clinical research: potential and pitfalls. BMJ.

[22] van Buuren S, Groothuis-Oudshoorn K (2011). Mice: multivariate Imputation by chained equations in R. J Stat Softw.

[23] Deng Jianxin SL, Deqiang H, Rui T (2019). Handling methods of missing data and their development trends. Stat Decis.

[24] Le VH, Kha QH, Hung TNK, Le NQK (2021). Risk score generated from CT-based radiomics signatures for overall survival prediction in non-small cell lung cancer. Cancers (Basel).

[25] Zeng Y, Cao W, Wu C (2022). Survival prediction in home hospice care patients with lung cancer based on LASSO algorithm. Cancer Control.

[26] Xu Y, Wang X, Huang Y, Ye D, Chi P (2022). A LASSO-based survival prediction model for patients with synchronous colorectal carcinomas based on SEER. Transl Cancer Res.

[27] Tibshirani R (1997). The LASSO method for variable selection in the Cox model. Stat Med.

[28] Xu S, Ross C, Raebel MA, Shetterly S, Blanchette C, Smith D (2010). Use of stabilized inverse propensity scores as weights to directly estimate relative risk and its confidence intervals. Value Health.

[29] Brookhart MA, Schneeweiss S, Rothman KJ, Glynn RJ, Avorn J, Stürmer T (2006). Variable selection for propensity score models. Am J Epidemiol.

[30] Austin PC (2014). The use of propensity score methods with survival or time-to-event outcomes: reporting measures of effect similar to those used in randomized experiments. Stat Med.

[31] Collins GS, Reitsma JB, Altman DG, Moons KGM (2015). Transparent reporting of a multivariable prediction model for individual prognosis or diagnosis (TRIPOD): the TRIPOD statement. Br J Surg.

[32] Sauerbrei W, Boulesteix AL, Binder H (2011). Stability investigations of multivariable regression models derived from low- and high-dimensional data. J Biopharm Stat.

[33] Venables WN, Ripley BD (2002). Statistics & Computing.

[34] Huang Y, Li W, Macheret F, Gabriel RA, Ohno-Machado L (2020). A tutorial on calibration measurements and calibration models for clinical prediction models. J Am Med Inform Assoc.

[35] Van Calster B, Wynants L, Verbeek JFM (2018). Reporting and interpreting decision curve analysis: a guide for investigators. Eur Urol.

[36] Kerr KF, Brown MD, Zhu K, Janes H (2016). Assessing the clinical impact of risk prediction models with decision curves: guidance for correct interpretation and appropriate use. J Clin Oncol.

[37] Cai M, Lin X, Wang X (2023). Ambient particulate matter pollution of different sizes associated with recurrent stroke hospitalization in China: a cohort study of 1.07 million stroke patients. Sci Total Environ.

[38] Lewsey J, Jhund PS, Gillies M (2010). Temporal trends in hospitalisation for stroke recurrence following incident hospitalisation for stroke in Scotland. BMC Med.

[39] Sun Y, Lee SH, Heng BH, Chin VS (2013). 5-year survival and rehospitalization due to stroke recurrence among patients with hemorrhagic or ischemic strokes in Singapore. BMC Neurol.

[40] Becker C, Zumbrunn S, Beck K (2021). Interventions to improve communication at hospital discharge and rates of readmission: a systematic review and meta-analysis. JAMA Netw Open.

[41] Kelly C, Hulme C, Farragher T, Clarke G (2016). Are differences in travel time or distance to healthcare for adults in global north countries associated with an impact on health outcomes? A systematic review. BMJ Open.

[42] Rao A, Barrow E, Vuik S, Darzi A, Aylin P (2016). Systematic review of hospital readmissions in stroke patients. Stroke Res Treat.

[43] Austin PC (2011). An introduction to propensity score methods for reducing the effects of confounding in observational studies. Multivariate Behav Res.

[44] Lin HJ, Chang WL, Tseng MC (2011). Readmission after stroke in a hospital-based registry: risk, etiologies, and risk factors. Neurology (ECronicon).

[45] Sun Y, Toh M (2009). Impact of diabetes mellitus (DM) on the health-care utilization and clinical outcomes of patients with stroke in Singapore. Value Health.

[46] Gentile NT, Vaidyula VR, Kanamalla U, DeAngelis M, Gaughan J, Rao AK (2007). Factor VIIa and tissue factor procoagulant activity in diabetes mellitus after acute ischemic stroke: impact of hyperglycemia. Thromb Haemost.

[47] Ribo M, Molina C, Montaner J (2005). Acute hyperglycemia state is associated with lower tPA-induced recanalization rates in stroke patients. Stroke.

[48] Khan MA, Schultz S, Othman A (2016). Hyperglycemia in stroke impairs polarization of monocytes/macrophages to a protective noninflammatory cell type. J Neurosci.

[49] Huang J, Liu B, Yang C, Chen H, Eunice D, Yuan Z (2013). Acute hyperglycemia worsens ischemic stroke-induced brain damage via high mobility group box-1 in rats. Brain Res.

[50] van Walraven C, Austin P (2012). Administrative database research has unique characteristics that can risk biased results. J Clin Epidemiol.

[51] Xu Y, Yang X, Huang H (2019). Extreme gradient boosting model has a better performance in predicting the risk of 90-day readmissions in patients with ischaemic stroke. J Stroke Cerebrovasc Dis.

