# Supplementary material for: Readmission After Ischemic Stroke in Ningxia, China, From 2017 to 2021: Retrospective Cohort Study
Source: Interact J Med Res. 2025 Jul 3;14:e67522. doi: 10.2196/67522 (PMC12244268; doi:10.2196/67522)

Supplemental Table 1. Correlation coefficient matrix between missing variable and other variables

|  | Admission route | NIHSS |
| --- | --- | --- |
| Sex | -0.00196 | 0.036042 |
| Age | 0.016397 | 0.021705 |
| Admission route | NA | 0.007361 |
| Diabetes | -0.00445 | -0.00558 |
| Thyroid disease | -0.0085 | 0.025287 |
| Dementia | -0.0031 | 0.027574 |
| Hypertension | -0.01496 | -0.01253 |
| Coronary heart disease | 0.018633 | 0.0206 |
| Heart failure | -0.00636 | 0.011961 |
| Acute upper respiratory tract infection | -0.00247 | 0.004029 |
| Paroxysmal tachycardia | -0.00583 | -0.0123 |
| Atrial fibrillation and flutter | -0.00542 | -0.01154 |
| Arteriosclerosis | 0.01916 | 0.046851 |
| Embolism and thrombosis | -0.00284 | 0.005833 |
| Pneumonia | 0.020266 | 0.024038 |
| Renal failure | -0.00272 | -0.01636 |
| Anemia | -0.00422 | 0.005428 |
| Transient ischemic attack and related syndrome | -0.00593 | 0.046731 |
| Urinary tract infection | -0.004 | -0.02631 |
| Parkinsonism | -0.00245 | 0.003738 |
| Treatment | -0.00503 | -0.104 |
| Length of hospital stay | -0.00753 | 0.036978 |
| NIHSS | NA | NA |

Supplemental Table 2. Characteristics between readmission and no readmission groups within 1 year

|  |  | | Cohort data | | | | SIPTW data | | | |
| --- | --- | --- | --- | --- | --- | --- | --- | --- | --- | --- |
|  |  | | no readmission | readmission | P | SMD | no readmission | readmission | P | SMD |
| n | |  | 9958 | 769 |  |  | 9958.0 | 768.5 |  |  |
| Sex (%) | | male | 5509 (55.3) | 444 (57.7) | 0.194 | 0.049 | 5501.9 (55.3) | 450.3 (58.6) | 0.102 | 0.068 |
|  | | female | 4449 (44.7) | 325 (42.3) | |  | 4456.2 (44.7) | 314.3 (40.9) |  |  |
| Age (%) | | <60 | 2360 (23.7) | 134 (17.4) | <0.001 | 0.258 | 2315.0 (23.2) | 176.9 (23.0) | 0.800 | 0.015 |
|  | | 60-69 | 2835 (28.5) | 190 (24.7) |  |  | 2808.2 (28.2) | 215.9 (28.1) |  |  |
|  | | 70-79 | 3143 (31.6) | 249 (32.4) | |  | 3148.7 (31.6) | 241.4 (31.4) |  |  |
|  | | ≥80 | 1620 (16.3) | 196 (25.5) | |  | 1686.1 (16.9) | 134.3 (17.5) |  |  |
| Admission route (%) | | emergency | 4846 (48.7) | 362 (47.1) | 0.612 | 0.035 | 4846.2 (48.7) | 360.7 (46.9) | 0.702 | 0.048 |
|  | | outpatient | 5098 (51.2) | 406 (52.8) | |  | 5097.8 (51.2) | 405.6 (52.8) |  |  |
|  | | transferred | 13 (0.1) | 1 (0.1) |  |  | 13.0 (0.1) | 2.1 (0.3) |  |  |
|  | | others | 1 (0.0) | 0 (0.0) |  |  | 1.0 (0.0) | 0.0 (0.0) |  |  |
| Parkinsonism (%) | | No | 9837 (98.8) | 754 (98.0) | 0.079 | 0.059 | 9836.2 (98.8) | 757.4 (98.6) | 0.573 | 0.019 |
|  | | Yes | 121 (1.2) | 15 (2.0) |  |  | 121.9 (1.2) | 11.0 (1.4) |  |  |
| Treatment (%) | | Anticoagulants | 9436 (94.8) | 739 (96.1) | 0.499 | 0.065 | 9438.5 (94.8) | 737.8 (96.0) | 0.551 | 0.059 |
|  | | Thrombectomy | 19 (0.2) | 1 (0.1) |  |  | 19.0 (0.2) | 1.0 (0.1) |  |  |
|  | | Thrombectomy and thrombolysis | 38 (0.4) | 2 (0.3) |  |  | 37.6 (0.4) | 2.0 (0.3) |  |  |
|  | | Thrombolysis | 465 (4.7) | 27 (3.5) |  |  | 462.8 (4.6) | 27.7 (3.6) |  |  |
| Anemia (%) | | No | 9579 (96.2) | 753 (97.9) | 0.014 | 0.102 | 9591.2 (96.3) | 735.3 (95.7) | 0.541 | 0.032 |
|  | | Yes | 379 (3.8) | 16 (2.1) |  |  | 366.9 (3.7) | 33.2 (4.3) |  |  |
| Thyroid disease (%) | | No | 8627 (86.6) | 661 (86.0) | 0.595 | 0.020 | 8626.3 (86.6) | 658.1 (85.6) | 0.486 | 0.029 |
|  | | Yes | 1331 (13.4) | 108 (14.0) | |  | 1331.8 (13.4) | 110.4 (14.4) |  |  |
| Dementia (%) | | No | 9761 (98.0) | 749 (97.4) | 0.237 | 0.042 | 9759.9 (98.0) | 748.4 (97.4) | 0.288 | 0.041 |
|  | | Yes | 197 (2.0) | 20 (2.6) |  |  | 198.5 (2.0) | 20.0 (2.6) |  |  |
| Transient ischemic attack and related syndrome (%) | | No | 9266 (93.1) | 710 (92.3) | 0.449 | 0.028 | 9264.5 (93.0) | 705.1 (91.8) | 0.243 | 0.048 |
|  | | Yes | 692 (6.9) | 59 (7.7) |  |  | 693.5 (7.0) | 63.4 (8.2) |  |  |
| Hypertension (%) | | No | 2895 (29.1) | 181 (23.5) | 0.001 | 0.126 | 2855.5 (28.7) | 224.0 (29.1) | 0.814 | 0.010 |
|  | | Yes | 7063 (70.9) | 588 (76.5) | |  | 7102.5 (71.3) | 544.5 (70.9) |  |  |
| Coronary heart disease (%) | | No | 7655 (76.9) | 557 (72.4) | 0.005 | 0.102 | 7623.9 (76.6) | 591.9 (77.0) | 0.775 | 0.011 |
|  | | Yes | 2303 (23.1) | 212 (27.6) | |  | 2334.2 (23.4) | 176.5 (23.0) |  |  |
| Paroxysmal tachycardia (%) | | No | 9267 (93.1) | 732 (95.2) | 0.024 | 0.091 | 9262.1 (93.0) | 730.9 (95.1) | 0.061 | 0.089 |
|  | | Yes | 691 (6.9) | 37 (4.8) |  |  | 696.0 (7.0) | 37.6 (4.9) |  |  |
| Diabetes (%) | | No | 7068 (71.0) | 507 (65.9) | 0.003 | 0.109 | 7053.6 (70.8) | 514.4 (66.9) | 0.035 | 0.084 |
|  | | Yes | 2890 (29.0) | 262 (34.1) | |  | 2904.5 (29.2) | 254.1 (33.1) |  |  |
| Atrial fibrillation and flutter (%) | | No | 9368 (94.1) | 723 (94.0) | 0.949 | 0.002 | 9359.4 (94.0) | 722.9 (94.1) | 0.931 | 0.004 |
|  | | Yes | 590 (5.9) | 46 (6.0) |  |  | 598.7 (6.0) | 45.5 (5.9) |  |  |
| Heart failure (%) | | No | 9170 (92.1) | 700 (91.0) | 0.296 | 0.038 | 9156.3 (91.9) | 712.5 (92.7) | 0.452 | 0.029 |
|  | | Yes | 788 (7.9) | 69 (9.0) |  |  | 801.7 (8.1) | 55.9 (7.3) |  |  |
| Arteriosclerosis (%) | | No | 4421 (44.4) | 304 (39.5) | 0.009 | 0.099 | 4409.0 (44.3) | 313.3 (40.8) | 0.089 | 0.071 |
|  | | Yes | 5537 (55.6) | 465 (60.5) | |  | 5549.0 (55.7) | 455.2 (59.2) |  |  |
| Embolism and thrombosis (%) | | No | 9787 (98.3) | 757 (98.4) | 0.746 | 0.012 | 9784.6 (98.3) | 758.7 (98.7) | 0.390 | 0.039 |
|  | | Yes | 171 (1.7) | 12 (1.6) |  |  | 173.4 (1.7) | 9.8 (1.3) |  |  |
| Acute upper respiratory tract infection (%) | | No | 9828 (98.7) | 761 (99.0) | 0.530 | 0.025 | 9827.3 (98.7) | 759.4 (98.8) | 0.781 | 0.012 |
|  | | Yes | 130 (1.3) | 8 (1.0) |  |  | 130.7 (1.3) | 9.0 (1.2) |  |  |
| Pneumonia (%) | | No | 9632 (96.7) | 743 (96.6) | 0.872 | 0.006 | 9627.5 (96.7) | 738.3 (96.1) | 0.465 | 0.033 |
|  | | Yes | 326 (3.3) | 26 (3.4) |  |  | 330.5 (3.3) | 30.2 (3.9) |  |  |
| Renal failure (%) | | No | 9804 (98.5) | 755 (98.2) | 0.555 | 0.021 | 9803.2 (98.4) | 754.4 (98.2) | 0.608 | 0.021 |
|  | | Yes | 154 (1.5) | 14 (1.8) |  |  | 154.8 (1.6) | 14.0 (1.8) |  |  |
| Urinary tract infection (%) | | No | 9618 (96.6) | 753 (97.9) | 0.047 | 0.082 | 9614.6 (96.6) | 753.3 (98.0) | 0.066 | 0.091 |
|  | | Yes | 340 (3.4) | 16 (2.1) |  |  | 343.5 (3.4) | 15.1 (2.0) |  |  |
| Length of hospital stay (%) | | <Q1(<8) | 2453 (24.6) | 103 (13.4) | <0.001 | 0.338 | 2372.6 (23.8) | 177.5 (23.1) | 0.820 | 0.018 |
|  | | Q1-Q2(8-10) | 2130 (21.4) | 161 (20.9) | |  | 2126.9 (21.4) | 166.5 (21.7) |  |  |
|  | | Q2-Q3(10-13) | 2940 (29.5) | 228 (29.6) | |  | 2940.8 (29.5) | 230.3 (30.0) |  |  |
|  | | >Q3(≥13) | 2435 (24.5) | 277 (36.0) | |  | 2517.7 (25.3) | 194.2 (25.3) |  |  |
| NIHSS (%) | | Q1(<=1) | 2568 (25.8) | 205 (26.7) | 0.015 | 0.123 | 2643.1 (26.5) | 200.8 (26.1) | 0.808 | 0.013 |
|  | | Q1-Q2(1-2) | 2898 (29.1) | 200 (26.0) | |  | 2902.8 (29.1) | 223.0 (29.0) |  |  |
|  | | Q2-Q3(2-4) | 2148 (21.6) | 186 (24.2) | |  | 2296.2 (23.1) | 180.9 (23.5) |  |  |
|  | | >Q3(>4) | 2344 (23.5) | 178 (23.1) | |  | 2115.9 (21.2) | 163.7 (21.3) |  |  |

Supplemental Table 3. Unweighted stepwise backward COX analysis

|  | HR | | Lower | Upper | P | |
| --- | --- | --- | --- | --- | --- | --- |
| 5 years |  | |  |  |  | |
| NIHSS Q1-Q2(1-2) | 0.84 | | 0.73 | 0.97 | 0.016 | |
| NIHSS Q2-Q3(2-4) | 0.96 | | 0.83 | 1.11 | 0.611 | |
| NIHSS >Q3(>4) | 0.82 | | 0.70 | 0.96 | 0.012 | |
| Length of hospital stay Q1-Q2(8-10) | 1.74 | | 1.43 | 2.12 | <0.001 | |
| Length of hospital stay Q2-Q3(10-13) | 1.99 | | 1.66 | 2.38 | <0.001 | |
| Length of hospital stay ≥Q3(≥13) | 2.64 | | 2.21 | 3.15 | <0.001 | |
| Urinary tract infection | 0.69 | | 0.49 | 0.99 | 0.041 | |
| Anemia | 0.49 | | 0.34 | 0.72 | <0.001 | |
| Pneumonia | 0.77 | | 0.57 | 1.04 | 0.093 | |
| Embolism and thrombosis | 0.72 | | 0.45 | 1.15 | 0.170 | |
| Atrial fibrillation and flutter | 0.83 | | 0.65 | 1.06 | 0.130 | |
| Paroxysmal tachycardia | 0.76 | | 0.60 | 0.98 | 0.032 | |
| Acute upper respiratory tract infection | 0.56 | | 0.31 | 1.01 | 0.053 | |
| Coronary heart disease | 1.10 | | 0.97 | 1.24 | 0.137 | |
| Hypertension | 1.30 | | 1.14 | 1.47 | <0.001 | |
| Age 60-69 | 1.32 | | 1.12 | 1.56 | 0.001 | |
| Age 70-79 | 1.54 | | 1.31 | 1.82 | <0.001 | |
| Age ≥80 | 1.97 | | 1.64 | 2.36 | <0.001 | |
| Sex woman | 0.83 | | 0.74 | 0.92 | 0.001 | |
| 1 year |  | |  |  |  | |
| NIHSS >Q3(>4) | | 0.82 | 0.67 | 1.01 | 0.056 |  |
| NIHSS Q2-Q3(2-4) | | 0.93 | 0.77 | 1.13 | 0.476 |  |
| NIHSS Q1-Q2(1-2) | | 0.76 | 0.63 | 0.92 | 0.005 |  |
| Length of hospital stay >Q3(≥13) | | 2.46 | 1.96 | 3.09 | <0.001 |  |
| Length of hospital stay Q2-Q3(10-13) | | 1.73 | 1.37 | 2.18 | <0.001 |  |
| Length of hospital stay Q1-Q2(8-10) | | 1.71 | 1.33 | 2.19 | <0.001 |  |
| Urinary tract infection | | 0.57 | 0.34 | 0.93 | 0.024 |  |
| Anemia | | 0.48 | 0.29 | 0.80 | 0.004 |  |
| Paroxysmal tachycardia | | 0.65 | 0.47 | 0.91 | 0.012 |  |
| Hypertension | | 1.26 | 1.06 | 1.49 | 0.008 |  |
| Diabetes | | 1.16 | 1.00 | 1.35 | 0.050 |  |
| Age ≥80 | | 2.24 | 1.80 | 2.80 | <0.001 |  |
| Age 70-79 | | 1.36 | 1.10 | 1.69 | 0.004 |  |
| Age 60-69 | | 1.15 | 0.92 | 1.44 | 0.214 |  |
| Sex woman | | 0.87 | 0.75 | 1.01 | 0.060 |  |

Supplemental figure 1. LASSO regression of group data on readmission within one year after SIPTW


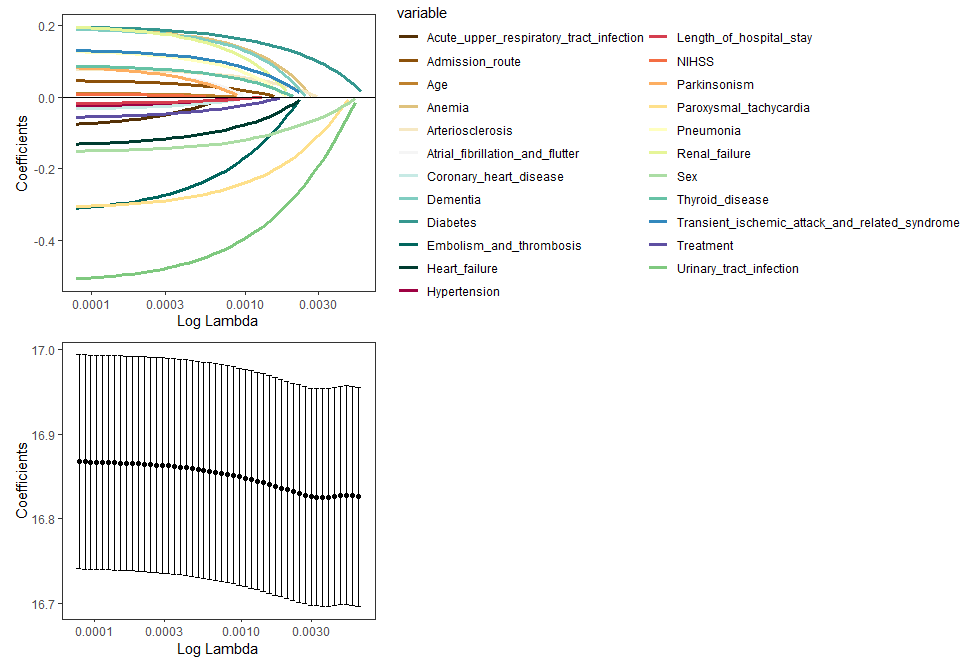

Supplement: Multimedia Appendix 1 [file ijmr-v14-e67522-s001.docx]
